# Plexiform Schwannoma of the Tongue in a Pediatric Patient with Neurofibromatosis Type 2: A Case Report and Review of Literature

**DOI:** 10.1155/2018/9814591

**Published:** 2018-10-15

**Authors:** Samir M. Amer, Aijan Ukudeyeva, Harold S. Pine, Gerald A. Campbell, Cecilia G. Clement

**Affiliations:** ^1^Department of Pathology, The University of Texas Medical Branch, Galveston, TX 77555, USA; ^2^Department of Otolaryngology, The University of Texas Medical Branch, Galveston, TX 77555, USA

## Abstract

**Introduction:**

Plexiform schwannoma is a rare variant of schwannoma that accounts for only 5% of all schwannomas. Herein, we present a rare case of plexiform schwannoma of the tongue in a pediatric patient with neurofibromatosis type 2 (NF2).

**Case Presentation:**

A 13-year-old female presented with a growing left-sided tongue mass. The patient has a past medical history of NF2. The tongue mass was excised and histopathological examination revealed a spindle cell tumor with multinodular growth pattern, with Verocay bodies' formation. Tumor cells were strongly positive for S-100 protein and negative for smooth muscle actin (SMA), and EMA highlighted perineural fibroblasts surrounding tumor nodules. These findings were diagnostic of plexiform schwannoma.

**Conclusion:**

Plexiform schwannoma of the tongue is an extremely rare tumor seen in patients with NF2. Clinical examination and histopathological evaluation are important for diagnosis of plexiform schwannoma.

## 1. Introduction

Schwannoma, also known as neurilemmoma, is a benign tumor of Schwann cells in the neural sheath of myelinated nerves throughout the body [1]. Up to 40% of all schwannomas are located in the head and neck region, of which 1% are found inside the oral cavity, frequently at the base of the tongue, although the tumor can occur anywhere else inside the mouth [1, 2]. Plexiform schwannoma is a rare variant of schwannoma, characterized by a multinodular (plexiform) intraneural growth pattern [3]. This variant accounts for only 5% of all schwannomas, with most cases occurring in the skin and subcutaneous tissue and showing a predilection for the head and neck region, similar to ordinary schwannomas [3, 4].

Neurofibromatosis type 2 (NF2), an autosomal dominantly inherited disease caused by structural mutation of the neurofibromin 2 (NF2) tumor suppressor gene on chromosome 22, is characterized by bilateral acoustic schwannoma in more than 90% of all patients. [1, 5]. Plexiform schwannoma usually presents as an isolated finding, and although it is unassociated with NF1, it may occur in the setting of NF2 and schwannomatosis [3, 6–10]. Malignant transformation in plexiform schwannoma has not been described, whereas plexiform neurofibroma carries a significant risk of malignant transformation, especially in NF1 patients, making this distinction important [9, 11]. Herein, we present a rare case of plexiform schwannoma of the tongue in a pediatric patient with NF2.

## 2. Case Presentation

A 13-year-old female presented to pediatric otolaryngology clinic for follow-up of a left-sided tongue mass, first detected in 2015, which was recently getting bigger and causing dental problems and difficulties with chewing. The patient has a past medical history of NF2, with bilateral acoustic neuromas (also known as vestibular schwannomas), diagnosed more than 10 years ago. During this period, she underwent removal of a right optic nerve glioma and several neurofibromas of the skin. She also has a strong family history of NF2, with her father and two sisters also diagnosed with NF2.

**Figure 1:**
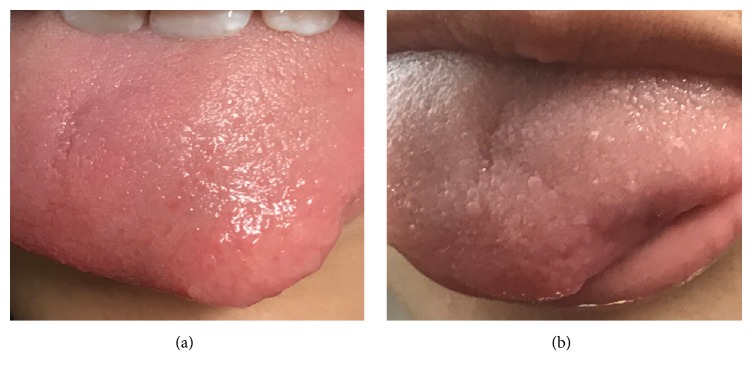
(a) Well circumscribed, 2 cm mass on left anterior tip of the tongue. (b) Tongue after excision of the mass.

Oral cavity examination revealed a well circumscribed mass on the left anterior tip of the tongue (Figure 1(a)). The mass was approximately 2.0 x 2.0 cm, nontender, and nonerythematous. Clinically, it appeared to be a tongue neural tumor associated with NF2.

Excisional biopsy of the tongue mass was performed under general anesthesia. The mass was removed with 2.0 mm margins around the lesion (Figure 1(b)). The postoperative period was uneventful. Gross examination of the specimen revealed a 1.7 x 1.4 x 0.5 cm nodule, which was serially sectioned to reveal multiple, well circumscribed white fibrous nodules ranging from 0.1 to 0.7 cm, with the largest nodule abutting the surgical margin.

**Figure 2:**
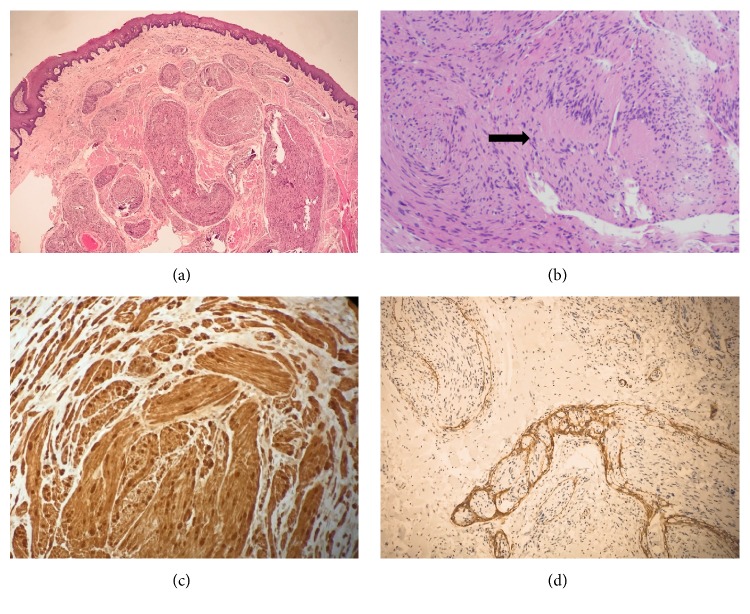
Histology of plexiform schwannoma. (a) Tumor cells forming interlacing fascicles and nodules (plexiform growth) (H&E, X20). (b) Cellular area with Verocay bodies (arrow), formed by palisading of nuclei and separated by cell processes of Schwann cells (H&E, X100). (c) Tumor cells are strongly and diffusely positive for S-100 (X100). (d) EMA-positive perineural fibroblasts surrounding nodules (X40).

Histopathological examination revealed a spindle cell tumor with multinodular growth pattern, resulting from interlacing fascicles of Schwann cells (plexiform growth) (Figure 2(a)). Most of the tumor consists of cellular areas (Antoni type A) with Verocay bodies, formed by palisading of nuclei and separated by cell processes of Schwann cells (Figure 2(b)). Immunohistochemical analysis revealed the tumor cells to be strongly positive for S-100 protein (Figure 2(c)) and negative for smooth muscle actin (SMA). EMA highlighted perineural fibroblasts surrounding tumor nodules (Figure 2(d)). These morphologic features, along with the immunoprofile, are diagnostic of plexiform schwannoma.

## 3. Discussion

Plexiform schwannoma usually presents as a single lesion in the skin and less commonly in the deep tissues [9, 12]. Harkin and Redd were the first ones to describe plexiform schwannoma in 1978, and since then very few cases have been reported.

Plexiform schwannoma occurs at any age but usually involves younger adults, being quite rare in children. Most plexiform schwannomas are solitary, sporadically occurring tumors, typically arising from superficial tissues (79%), occurring most commonly in the head and neck region (23%), and account for 15% of cutaneous schwannomas [3, 13].

Oral lesions of all types are present in about 75% of patients with NF1, in which the tongue is the most common location, while oral manifestations among NF2 cases are extremely rare [14, 15]. The case reported in this article, plexiform schwannoma in a 13-year-old with NF2 presenting with a 3-year history of slowly progressive mass on the tip of the tongue, fits in the latter category. A review of the literature over the past 30 years yielded only five cases, including the current one, of patients with NF2 having oral manifestations with a single, localized lesion on the tongue [14–17]. Only one of these cases was diagnosed as schwannoma, the other 2 were amyloid tumors, and one was clinically suspected to be a neurofibroma. Therefore, our case is, to the best of our knowledge, the second reported in the literature.

Tongue schwannoma presents as a painless slowly growing mass and is usually asymptomatic unless enlarged in size [18–21]. Nisa et al. [22] reported a giant plexiform schwannoma of the tongue base in a patient with a 20-year history of a slowly enlarging tongue mass. The reported size of 8.5 x 5.0 x 6.0 cm for that mass makes it the largest tongue schwannoma documented so far. That patient only sought intervention after experiencing progressive dysphonia and severe dysphagia. Although our patient was diagnosed with the tongue mass 3 years ago, she only sought intervention after having chewing problems and discomfort.

Given the child's presentation of a slowly growing mass of the tongue, a wide range of benign soft tissue lesions should be considered as differentials. Lingual schwannoma, neurofibroma, amyloid tumor, lipoma, hemangioma, lingual thyroid, cystic lesions such as dermoid cysts and mucoid cysts, and benign salivary gland tumors are some examples of many on the list [23, 24]. As previously mentioned, it is important to differentiate schwannoma from neurofibroma, another benign nerve sheath tumor found in patients with NF1 and rarely in NF2, since the latter implies a higher risk of malignant transformation. Approximately 15% of patients with neurofibromatosis will have malignant transformation in one or more neurofibromas, in marked contrast to the typical behavior of schwannomas [23, 25].

The diagnosis of plexiform schwannoma relies on the characteristic gross and histopathologic examination that demonstrates a multinodular growth pattern of predominantly hypercellular Antoni A areas consisting of fusiform Schwann cells with a typical palisading pattern around central eosinophilic areas, known as Verocay bodies [26]. In our patient, histologic examination of the mass showed these characteristic patterns.

Conventional and plexiform schwannomas can be differentiated from other nerve tumors by using immunohistochemical staining, where schwannoma shows strong, diffuse S-100 staining, a membrane protein currently considered the most sensitive marker for tumors originating from Schwann cells [27]. Given the strong immunoreactivity to S-100, negative staining for SMA, and the characteristic histologic pattern, a diagnosis of plexiform schwannoma was confirmed in our patient.

Transoral excisional surgery is the treatment of choice of oral plexiform schwannoma [28]. However, for basal tongue schwannoma, cervical approach (transhyoid or submandibular) is preferred for better visualization and removal. Impairment of oral function and discomfort are the main indications for removal of tongue schwannoma as in our presented case. Follow-up of our patient showed no signs of recurrence up to the present time. The rate of local recurrence is extremely low after complete resection with free surgical margins, although recurrence is possible with incomplete excisions [3].

## 4. Conclusion

Plexiform schwannoma is a benign tumor of Schwann cells, very rarely seen in patients with NF2. Clinical examination and histopathological evaluation are important for diagnosis of plexiform schwannoma. Surgical removal is the best treatment for these tumors, with extremely low recurrence rate.

## Data Availability

Our conclusions arise from the evaluation of the histopathologic findings and selected clinical data described in this study. No other data can be released due to patient confidentiality.
